# From Natural Products to Small Molecules: Recent Advancements in Anti-MRSA Therapeutics

**DOI:** 10.1021/acsmedchemlett.5c00061

**Published:** 2025-03-14

**Authors:** Jacqueline R. Smith, Andrew R. LeBlanc, William M. Wuest

**Affiliations:** Department of Chemistry, 221304 Emory University, Atlanta, Georgia 30322, United States

**Keywords:** MRSA, Pleuromutilin, Carbazoles, Repurposed drugs, Flavonoids, Retinoids

## Abstract

The urgent need for unique small molecules to treat increasing resistance in gram-positive pathogens, particularly methicillin-resistant *Staphylococcus aureus*, has motivated several creative research endeavors over the past decade. Recent advances have been inspired by natural products such as pleuromutilin, discovered in high-throughput screens, or repurposed approved drugs like sorafenib. This microperspective spotlights bioactive compounds, ranging from natural products to small molecule scaffolds, that have been reported in recent literature, highlighting their mechanisms of action, structure–activity relationships, and future potential.

## Graphical abstract

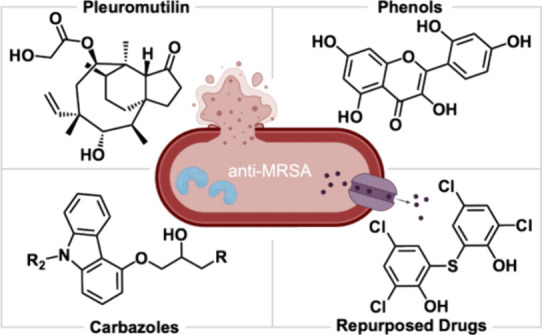

The development of new gram-positive pathogen therapeutics has historically been overlooked. Often considered an “easier” antimicrobial target due to its single membrane nature and lack of lipopolysaccharides, gram-positives have been surpassed in priority to their gram-negative counterparts. Comparatively, gram-negative bacteria have an outer membrane, containing a peptidoglycan layer in periplasmic membrane and then a cytoplasmic membrane. This additional membrane in gram-negative bacteria is highly selective, making antibiotic development difficult. While treatment of multidrug-resistant gram-negative pathogens presents a challenge, development of alternative gram-positive agents is equally important due to both their propensity to develop resistance and the societal impact that these infections have overall.

The resistance mechanisms in bacteria can be summarized into five approaches (Figure 1A). Bacteria can modify antibiotics through (i) structural alteration or (ii) complete degradation, resulting in an ineffective antibiotic. Intercellular concentration of antibiotic can be limited by (iii) decreased cellular uptake or (iv) antibiotic efflux. (v) Protein modification of an antibiotic target can prohibit the antibiotic from binding to its target.^1^ The widespread occurrence of these mechanisms is the driving force for antimicrobial resistance (AMR).^2^ At the center of the AMR crisis is *Staphylococcus aureus*, a gram-positive bacterium that causes a wide range of infections. The United States Centers for Disease Control and Prevention (CDC) has deemed *S. aureus* a serious threat.^3^ *S. aureus* resistance to β-lactams, which inhibit cell wall synthesis, was first detected as early as 1942.^3^ The ineffectiveness of penicillin motivated the development of methicillin; it was introduced to the clinic in 1959, and resistance was detected only one year later (Figure 1B).^3,4^ Methicillin-resistant *S. aureus* (MRSA) has caused 323,700 hospitalizations, over 10,000 deaths, and has been estimated to attribute a total of $1.7 billion to healthcare costs in 2017.^2^ MRSA resistance to β-lactams is imparted by penicillin binding protein 2a (PBP2a), a transpeptidase that has affinity for β-lactams, thus allowing cell wall synthesis to continue.^5^

**Figure 1:**
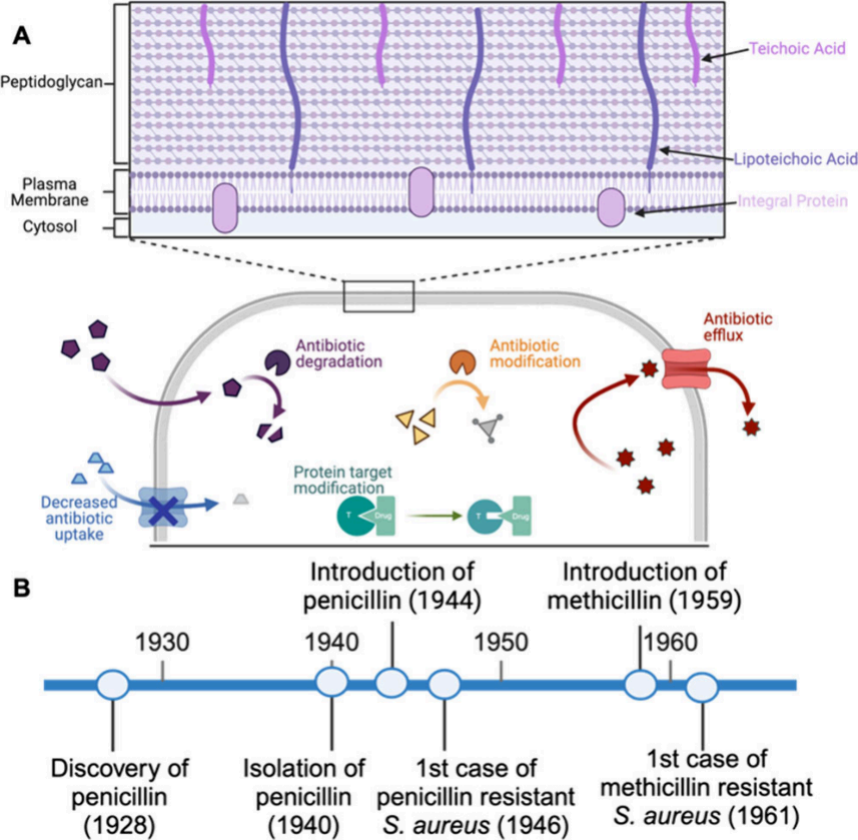
(A) Structure of gram-positive bacterial membranes, and cartoon of antibiotic resistance mechanisms. (B) Timeline of antibiotic and resistance development for MRSA.

Recent studies have attempted to address these resistance mechanisms through unique and strategically designed small molecules. Three scaffolds that have gained recent attention are pleuromutilin, carbazole, and phenolic compounds. Pleuromutilin is a diterpene natural product, and semisynthetic analogs have displayed high affinity for the ribosome of MRSA, inhibiting protein synthesis.^6^ Carbazole-based compounds have been found to increase bacterial cell membrane permeability.^7^ They share a mechanism of action with several phenolic compounds, particularly derivatives of natural products honokiol and magnolol, plant flavonoids, and synthetic retinoids.^8,9^ Beyond these three motifs, there has been success in repurposing drugs to become potent antibiotics.^10^ This microperspective aims to highlight these advancements in anti-MRSA compounds, specifically focusing on structure activity relationships (SAR), creative modifications, promising scaffolds, and areas of improvement.

## Pleuromutilin

Diterpene natural product (+)-pleuromutilin (Figure 2), discovered in 1951, is produced by the fungi *Pleurotus mutilus* and *Pleurotus passeckerianus*.^11^ Pleuromutilin’s moderate antimicrobial activity against Gram-positive bacteria is imparted by inhibition of bacterial protein synthesis. The scaffold binds to the peptidyl transferase center (PTC) of the 50S subunit of the ribosome. Elucidation of the binding mode, through X-ray cocrystallography, shows that the tricyclic core binds to the A-site of the PTC, while the C14 chain projects into the P-site, where tRNA specifically associates.^6^ This directly hinders tRNA association and therefore inhibits peptide bond formation.^11^ The unique mechanism of pleuromutilin, and its synthetic analogs, is credited to limiting the rate of resistance development.^12^ Medicinal chemists have taken advantage of this well-elucidated mechanism to derivatize the C14 side chain of pleuromutilin in an effort to improve activity.

To date, four semisynthetic analogs featuring C14 side chain modifications have been approved by the US Food and Drug Administration (FDA) (Figure 2B).^13–16^ Tiamulin and valnemulin were approved for veterinary use in 1979 and 1999, respectively.^13,14^ Retapamulin was the first pleuromutilin derivative approved for short-term human use to treat skin and soft tissue infections in 2007.^15^ Lefamulin was approved in 2019 by the FDA for treatment of community-acquired pneumonia through oral and intravenous administration.^16^ However, a high oral dose (600 mg) is required to impart the full oral absorption and bioavailability potential of lefamulin.^17^ This undesirable dosage, as well as some off-target effects that lead to contraindication for patients with arrhythmic conditions,^17^ have inspired the search for new pleuromutilin analogs with more favorable properties.

**Figure 2:**
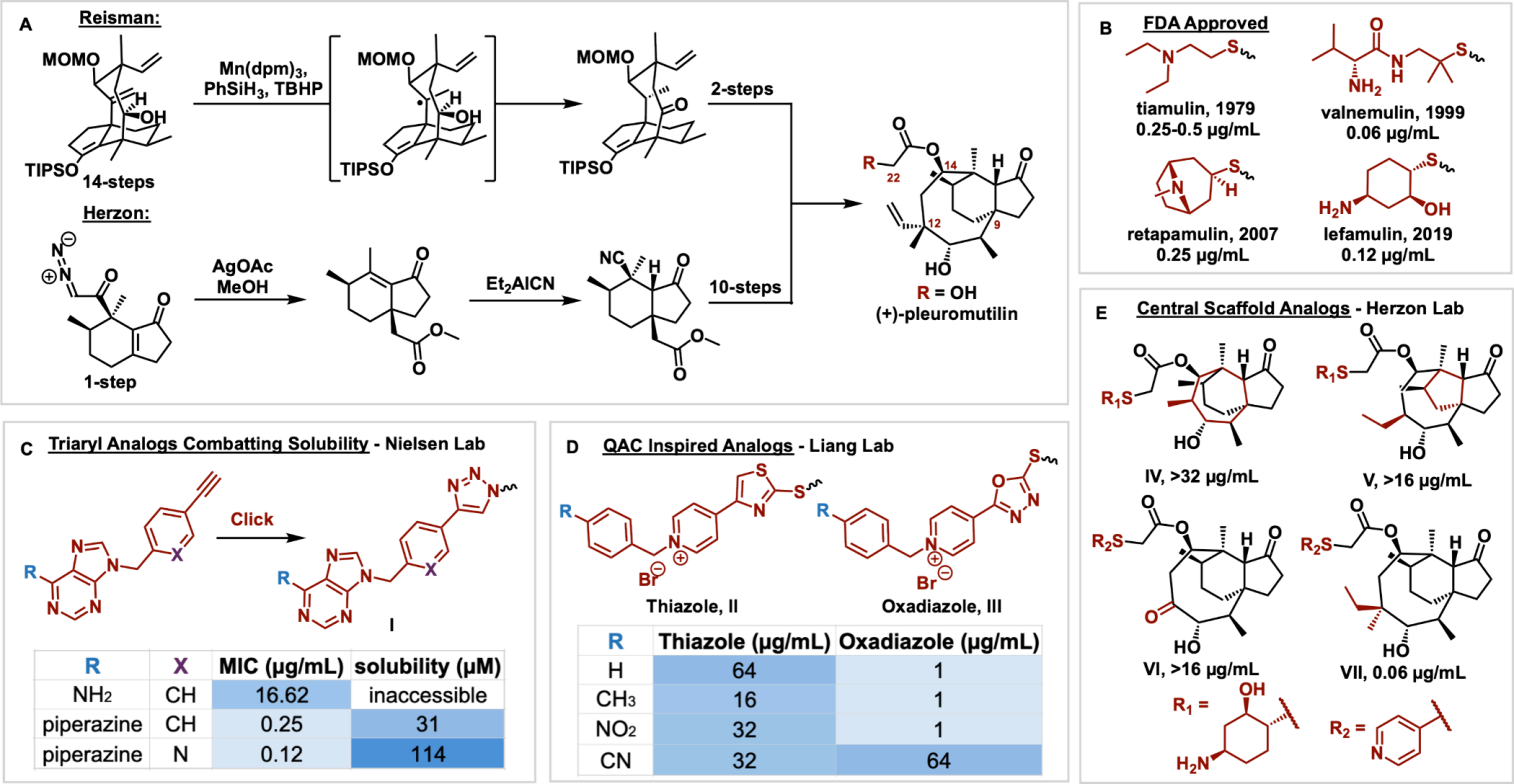
(A) Structure of pleuromutilin with key steps from the Reisman and Herzon syntheses. (B) FDA approved pleuromutilin-based drugs. (C) Nielsen Lab pleuromutilin analogs optimizing solubility. (D) Liang Lab analogs featuring QACs. (E) Herzon Lab analogs featuring skeletal modifications of pleuromutilin.

Both the Herzon and Reisman laboratories have completed total syntheses of pleuromutilin (Figure 2A).^18^ The Herzon synthesis leverages a Nagata hydrocyanation, fragment coupling with an organolithium and enimide, and a stereoselective reductive cyclization culminating in a 19 step total synthesis.^18^ Seven months later, the Reisman lab published an 18 step synthesis, featuring a SmI_2_-mediated cyclization and stereoselective transannular [1,5]-hydrogen atom transfer.^19^ These syntheses unlock a wider SAR scope that would be otherwise inaccessible. The Herzon lab took advantage of their synthetic method to make derivatives of the pleuromutilin skeleton, discussed below.^20^ However, chemistry on the C14 side chain is broadly tolerated by the rest of the pleuromutilin scaffold, and derivatization at this site is typically beneficial to activity due to the C14 side chain’s position in the tRNA binding site. Thus, despite these published methods, commercially available (+)-pleuromutilin is widely used as the synthetic starting point for a majority of medicinal chemistry campaigns.^21–26^

The C22 alcohol of the pleuromutilin scaffold is the most common site for derivatization. A thioether linker is commonly utilized for its enhanced antibacterial activity.^24–27^ Aryl substitutions of the thioether are found to increase antibacterial activity,^21–27^ likely due to a stacking interaction with U2585 in the binding pocket.^28^ This is a new structural motif not found in the four currently approved pleuromutilin derivatives. Triazoles and thiazoles, along with other 5-membered heterocycles, showed increased bacterial activity.^21–23,25^ For example, the Nielsen lab has identified a triaromatic pleuromutilin conjugate featuring a 1,2,3-triazole moiety.^21,22^ 1,2,3-triazole synthesis with copper-catalyzed alkyne–azide cycloaddition, or “click” chemistry, allows for late stage diversification, enabling biological evaluation of multiple functional groups.^21,22^ Lead compound **I** exhibits activity on par with lefamulin but a piperazine substituent shows more favorable absorption, distribution, metabolism, excretion, and toxicity (ADMET) properties not found in lefamulin (Figure 2C).^21,22^ Lefamulin also possesses disfavored off-target effects for the human ether-related-a-go-go (hERG) encoded potassium channel that are not observed in the Nielsen compounds.^21,22^

The Liang lab has creatively coupled the potent activity of pleuromutilin with the known antibacterial properties of quaternary ammonium compounds (QACs).^25^ QACs are common disinfectants used industrially and in household products, and utilize their positively charged quaternary nitrogens to bind to anionic phospholipids in bacterial cell membranes, altering permeability.^25,29^ Unfortunately, several human pathogens are developing resistance genes to QACs.^29^ However, pleuromutilin derivatives show low rates of resistance development.^25^ Quaternized centers also aid the otherwise poor aqueous solubility found in most pleuromutilin compounds.^25^ The lead pleuromutilin-QAC analogue **II** features a thiazole moiety with a substituted pyridine providing the quaternary center (Figure 2D). Compound **II** has an 8-fold increase in potency compared to that of moxifloxacin and significantly improved solubility.^25^ No significant cytotoxicity against human cells was detected, suggesting the compounds are selective for bacteria. It is proposed that these compounds both degrade the cell membrane integrity as well as interact with the PTC of the ribosome.^25^ These factors contribute to a higher survival rate of mice with MRSA over valnemulin (**II**, 5 mg/kg: 57.14%, valnemulin, 5 mg/kg: 42.86%).^25^

Unlike these previous examples, the Herzon group’s synthetic approach toward pleuromutilin analogue development enables structural diversification beyond the C14 side chain.^20^ An unexpected Wolff rearrangement, with broad substrate tolerance, gave access to the C9 quaternary center (compounds **IV–VII**, Figure 2E).^20^ This reaction enabled other bicyclic intermediates that led to novel pleuromutilin structures featuring different skeletal rings (compounds **IV–V**).^20^ These analogues were nearly inactive in MRSA, but the extensive synthetic efforts did result in a compound, featuring C12 modifications, with activity comparable to that of lefamulin.^20^ This study emphasizes the specific affinity the pleuromutilin scaffold contains for the PTC of the ribosome, as well as demonstrates the greater SAR scope that a fully synthetic approach enables.

## Phenolic Compounds

### Honokiol and Magnolol

Honokiol and magnolol, two phenolic natural products, have recently gained medicinal attention due to their under explored activity against gram-positive pathogens. Honokiol and magnolol are diphenolic isomers extracted from *Magnolia officinalis*, and their medicinal properties date back to traditional Chinese medicine.^8,30^ Magnolol has activity against *S. aureus* and MRSA, while honokiol exhibited selective, albeit modest, anti-MRSA activity (honokiol, 10–50 μg/mL; magnolol, 20 μg/mL).^8^ They were found to be synergistic with several antibiotics, including oxacillin, ampicillin, chloramphenicol, tetracycline, and cefoxitin (magnolol/oxacillin, 16 μg/mL/50 μg/mL; honokiol/oxacillin, 23 μg/mL/50 μg/mL).^8^ Expression of the *mecA* gene, responsible for the induction of PBP2a production, was inhibited in a dose-dependent manner by magnolol and honokiol, thus restoring β-lactam activity.^8^ Furthermore, magnolol and honokiol have complete restorative effects for aminoglycosides.^30^ Both natural products can remove preformed *S. mutans, S. aureus,* and MRSA biofilms. Our group identified that these compounds, and their derivatives, lead to lysis of gram-positive bacterial cells, confirmed with propidium iodide (PI) assays and transmission electron microscopy (TEM).^31^

Recently, chemists have utilized the magnolol/honokiol scaffold as a starting point for medicinal chemistry campaigns.^32–35^ Our group in collaboration with the Kozlowski lab developed honokiol analogues to combat gram-positive pathogens in the oral microbiome.^36^ The Guo and Liu groups have utilized cationic antimicrobial peptide (CAMP) mimics to decorate the natural product due to the suspected cell membrane-related activity of honokiol (compound **VIII**, Figure 3A). While CAMPs contain bactericidal properties with low probability of resistance, they have poor pharmacokinetic profiles and poor *in vivo* activity.^37,38^ Substituting the phenolic moieties with quaternary ammonium and amide groups showed improved MIC (0.5–1 μg/mL) against 10 MRSA strains.^32^ These compounds displayed rapid bactericidal ability and were less likely to induce drug resistance. Furthermore, they were found to significantly reduce viable biofilm counts at a concentration of 64 μg/mL.^33^ DiSC3(5) (3,3′-dipropylthiadicarbocyanine iodide), DAPI/PI (4′,6-diamidino-2-phenylindole), and SYTOX Green fluorescent probe assays conclude that the lead targeted the cell membrane and altered cell permeability.^33^ Furthermore, CAMP mimic substitution on magnolol, rather than honokiol, exhibited comparable antibacterial *in vivo* effects to vancomycin at the same dose.^33^

**Figure 3:**
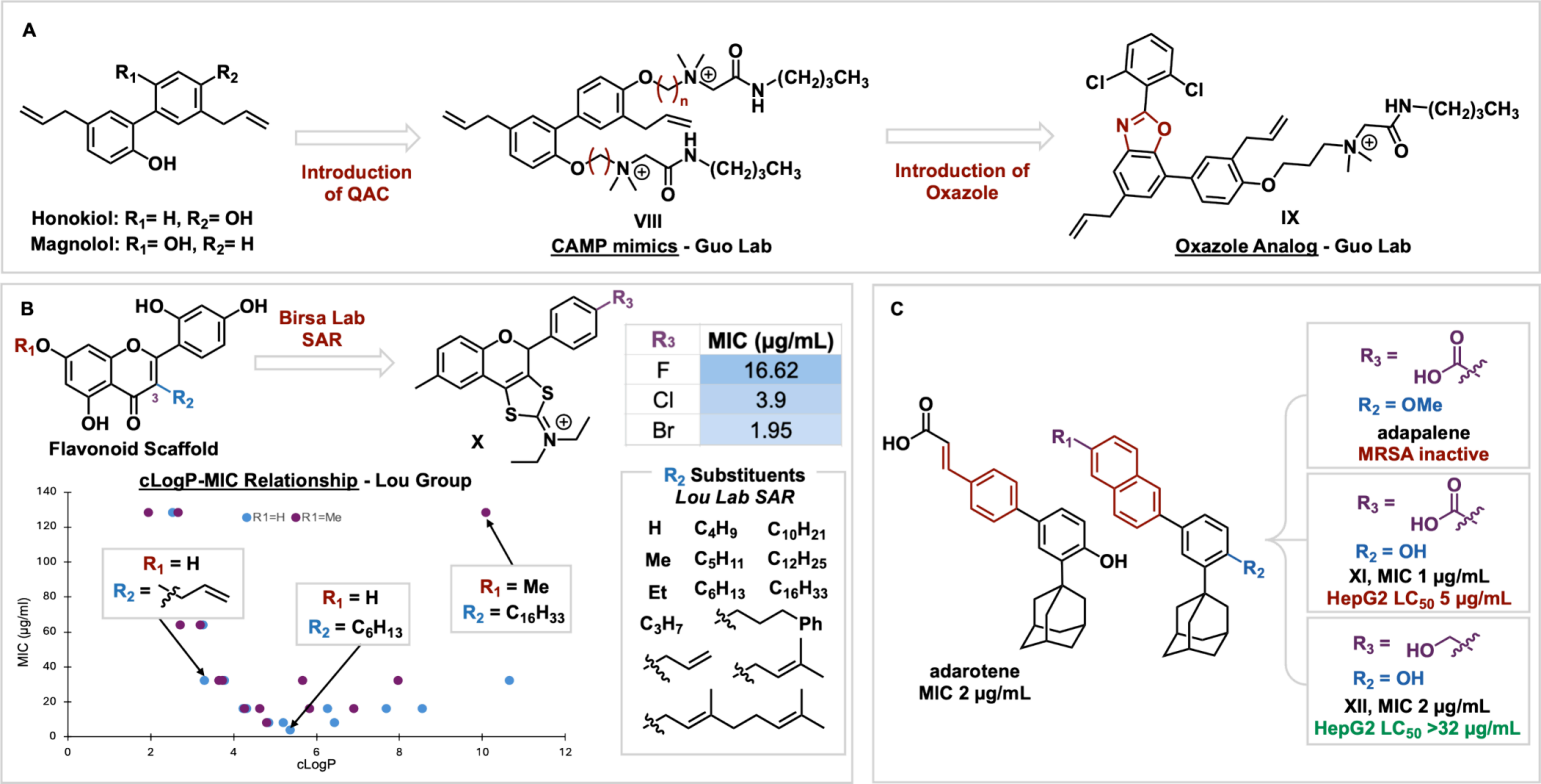
(A) Honokiol and magnolol and related derivatives from the Lio and Guo Laboratories. (B) Flavonoid derivatives from the Birsa Lab, and the cLogP relationship identified from the Lou Lab. (C) Retinoids, adarotene and adapalene, and derivatives from the Wuest Lab.

Another analogue, compound **IX**, by the Guo group replaced the phenol with an oxazole moiety.^34^ An oxazole is a feature of many commercialized antimicrobials, and may impart enhanced *in vivo* activity as they are metabolically stable bioisosteres of esters and amides.^34,39^ To further verify the compound’s membrane disruption mechanism, the Guo group utilized an exogenous addition method to identify which phospholipid component of the cell membrane serves as the target.^34^ The compound’s MIC increased as external concentrations of phosphatidylglycerol and cardiolipin were increased, implying the compound specifically interacted with these components of the membrane.^34^

### Flavonoids

Flavonoids, characterized by their polyphenolic structure, are well-known for their bactericidal effects.^40^ They are the largest group of plant-derived natural products and have been used therapeutically since ancient times. Twenty-seven types of flavonoids have been identified to have anti-MRSA activity.^41^ Most natural flavonoids disrupt the cell membrane and, in some cases, even display moderate efflux pump inhibitory activity.^9^ They have also been found to inhibit biofilm formation, reverse antibiotic resistance, act synergistically with antibiotics, and decrease virulence factors.^42^ Investigators have looked into synthetic flavonoids for their increased potency and more favorable ADMET properties.^43–45^

The Birsa lab has synthesized flavonoid-derived structures that exhibit promising anti-MRSA activity.^46^ The tricyclic scaffold of compound **X** features a unique 1,3-dithiolium-2-yl structure, confirmed by X-ray analysis.^46^ Not only was their lead gram-positive specific, but displayed excellent bactericidal and bacteriostatic effects against MRSA.^46^ Fluorescent PI probes were used to determine that the tricyclic flavonoid compromised the integrity of the cell membrane.^46^ The Luo group synthesized 63 flavonoid derivatives informed by a screen of 162 natural flavonoids.^47^ This screen identified the importance of lipophilic groups at the C3 position.^47^ Based on the membrane disrupting mechanism, the group incorporated amphiphilic moieties to further enhance affininty.^47^ The Luo group identified a relationship between clogP and antibacterial activity (Figure 3B). Specifically, they found that compounds exhibited most potent activity when clogP was between 5.0 and 7.6.^47^ Their lead compound showed bacteriostatic and bactericidal effects, and both eradicated and inhibited biofilm. Furthermore, this lead compound showed a slow rate of resistance.^47^ This success translated *in vivo* with the flavonoid analogue giving better therapeutic effects than vancomycin for MRSA skin infections.^47^

### Retinoids

Retinoids, comprising of vitamin A and its natural and synthetic derivatives, have a rich history of treating skin ailments and infections through both oral and topical administration.^48,49^ For example, synthetic retinoid adapalene was FDA approved in 1996 for the treatment of acne.^49^ Natural retinoid retinaldehyde is used as a popular dermatological agent, but was also found to possess potent anti-MRSA activity (MIC 1–16 μg/mL, 20 strains).^50^ Our group identified retinoid **XI** out of 82,000 synthetic small molecules in an established high throughput *Caenorhabditis elegans*-MRSA infection screen.^51^ **XI** was selected for both its excellent activity and structural similarity to known therapeutic adarotene (Figure 3C).^51^ Notably these compounds were also active against staphylococci isolates from ocular infections.^52^ *S. aureus* was unable to develop significant resistance to these compounds compared to ciprofloxacin or daptomycin.^51^ Modest resistance was manifested in genes *graS*, *yjbH*, and *manA*, all membrane related.^51^ Indeed, a SYTOX Green assay confirmed these compounds act through membrane permeabilization.^51^ Moreover, the new synthetic analogues were effective against MRSA persister cells, defined later.^51^ A simplified analogue **XII** was shown to maintain activity while showing improved hemolytic activity and less cytotoxicity.^51^ Molecular dynamics simulations indicate that two polar branched groups (the primary alcohol/carboxylate and the phenol) are necessary for attachment to lipid heads of the bacterial membranes.^51^

## Carbazoles

The carbazole scaffold can both interact with bacterial membranes^7^ and, depending on functionalization, interfere with specific targets;^53^ these complementary modes of action make the carbazole an attractive starting point for medicinal chemistry campaigns.^37,38,53–55^ Most recent carbazole-based SAR studies either start from screens,^7,54,56^ or are inspired by the antibiotic reputation of carbazoles.^37,38,53,57^ Interestingly, a series of carbazole alkaloid natural products isolated from *Clausena wallichii* roots, clausenawallines C–F, exhibit significant activity against MRSA, but are typically not credited for inspiring carbazole-based anti-MRSA investigations.^58^ Carbazoles are also commercially available with well-defined chemical processes, enabling thorough medicinal chemistry campaigns.^59^ There are currently no carbazole-based antimicrobial agents approved for clinical use, presenting a unique opportunity for investigation.^37^

The Liu group has focused on substituting carbazoles with amphiphilic structures to biomimic the effect of CAMPs.^37,38^ Carbazole’s reputation of strong drug-like properties compensated for the poor pharmacokinetic profiles of CAMPs.^37,38^ Thus, the group designed a series of carbazole-based compounds featuring CAMP bioisosteres.^37,38^ These amphiphilic isosteres strengthen the interaction between cationic carbazoles and the anionic membrane, as well as introduce hydrophobicity to interact with bacterial membrane phospholipid bilayers.^37,38^ This promotes formation of pores in the membrane, leading to cell death.^37,38^ Incorporation of QACs elicits the same effect. The structure also features a prenyl group, a feature of several plant flavonoids with activity against MRSA (Figure 4).^40^ Inspired by their initial results, the group incorporated chelating groups (dipicolyamine) and metal cations (Zn^2+^), thus increasing cationic character and further enhancing anionic membrane affinity.^38^

**Figure 4:**
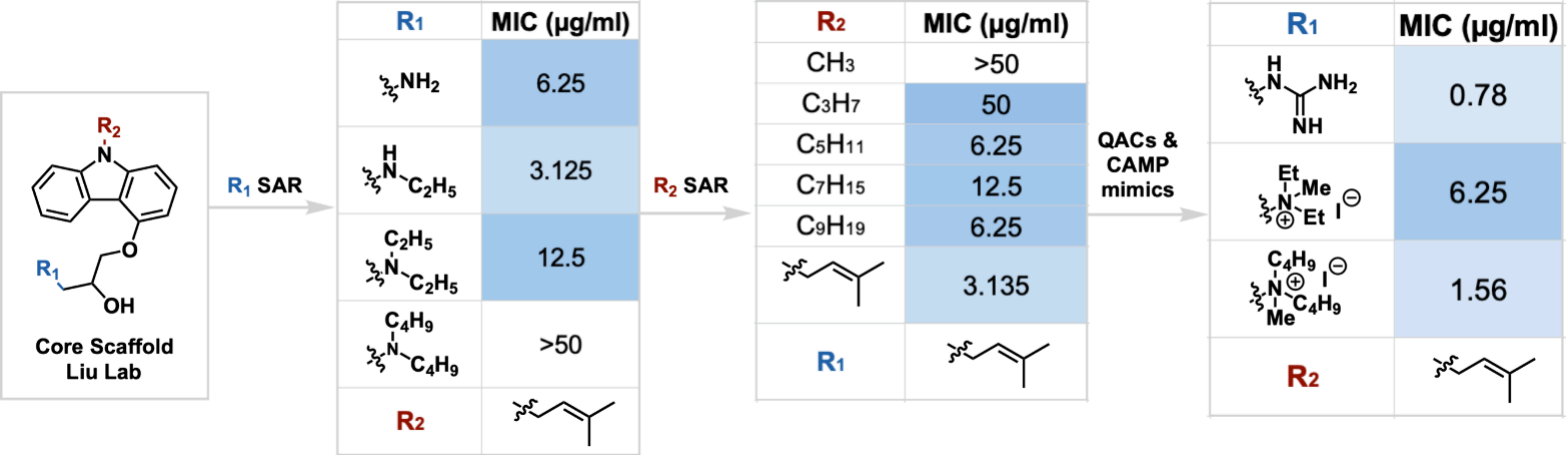
SAR of the carbazole-based analogs from the Liu Lab.

The Zhou lab has designed a series of carbazole-oxadiazoles with rapid bactericidal activity equivalent to or stronger than vancomycin.^53,57^ The series is *N*-substituted with an oxadiazole-thiol. In an effort to understand the antibacterial mechanism of these compounds, fluorescent probes were utilized to measure membrane damage, in which a dose-dependent response to compound exposure was found, confirming the lead increased the permeability of the membrane.^53^ Another dose-dependent response was detected when measuring the leakage of cytoplasmic content from treated cells.^53^ They were found to have no serious cytotoxicity against Hek 293T and HeLa cells, low hemolytic effects to human red blood cells, and no serious *in vivo* toxicity to mice.^53^ These studies imply that a carbazole scaffold can selectively damage the bacterial cell membrane.

Tsou and Shia groups conducted a screen and identified an indole to have modest anti-MRSA activity. Inspired by previously observed carbazole activity, SAR studies led to a lead containing a carbazole core, generating an 8-fold increase in anti-MRSA activity.^54^ Several groups, however, have identified indoles to possess potent antibacterial activity. Muller and Kalscheuer groups made bisindole alkaloid compounds with strong MRSA membrane-permeabilizing activity.^60^ Indolinones developed in the Sieber group displayed significant *S. aureus* activity,^61^ and the Melander group has made significant efforts in developing aminoimidazoles.^62^ However, both of these examples involve different mechanisms of action from membrane disruption by carbazoles.^61,62^

## Repurposed Drugs

Despite these successes with natural product and small-molecule-based scaffolds, the mechanisms of attacking membrane synthesis and integrity, DNA synthesis, and protein synthesis are already known to bacteria, and have already amassed several resistance mechanisms.^10^ Moreover, there is a large amount of chemical space with untapped anti-MRSA potential.^10^ Repurposing approved or investigational drugs for activity against MRSA presents an opportunity to investigate unique mechanisms that bacteria have not yet developed resistance toward.^10^ Efforts toward repurposing known compounds are not only met with less toxicity but can also be more time- and cost-effective.^10^ Repurposing well-investigated or FDA-approved compounds results in low-risk investigation, as they are typically already proven to be safe.^10^

Our lab in collaboration with the Mylonakis group has successfully repurposed several drugs to combat MRSA. Nonthiazolidinedione PPARγ partial agonist (nTZDpa) was identified in a high-throughput screen, and has been investigated *in vivo* for diabetes therapy (Figure 5).^63^ Not only is nTZDpa and its analogues highly effective against MRSA, but it is also synergistic with aminoglycosides and active against MRSA persisters.^63^ Persisters are bacteria in a metabolically low-energy state, showing reduced biosynthetic processes, resulting in antibiotic tolerance in many cases.^64^ They are particularly abundant in biofilms and cause chronic and relapsing infections.^64^ A SYTOX Green assay confirms nTZDpa induced membrane permeabilization.^63^ nTZDpa bares a strong structural similarity to Tsou and Shia’s hit compound, which through SAR resulted in a carbazole lead.^54^ Furthermore, both their carbazole and nTZDpa share a mechanism of action.^54,63^

**Figure 5:**
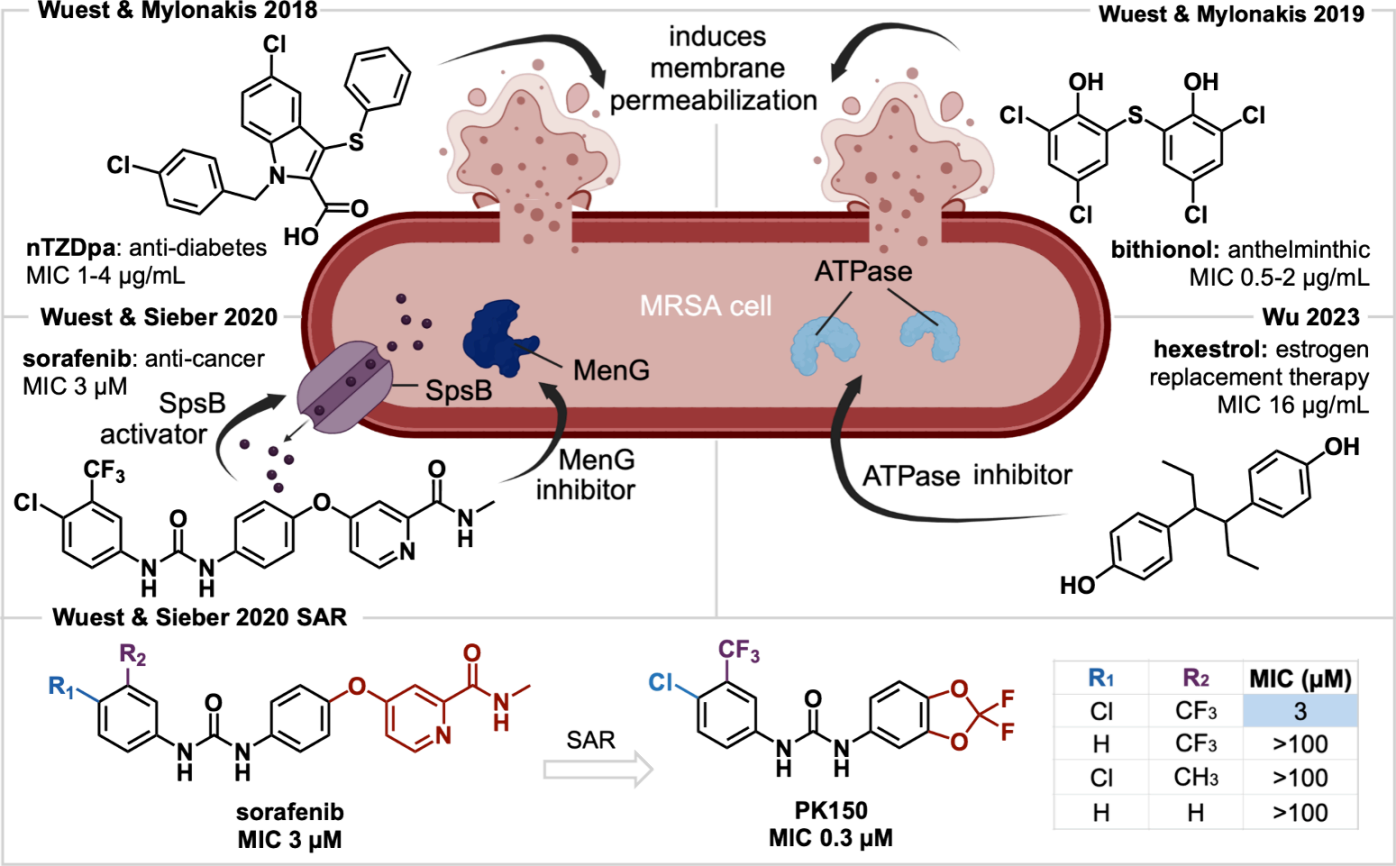
Repurposed drugs nTZDpa, bithionol, sorafenib, and hexestrol and their modes of action. Sorafenib SAR to give lead compound **PK150** is highlighted.

Our lab, again in collaboration with the Mylonakis group, also identified bithionol, a clinically approved anthelminthic drug used primarily in veterinary medicine, to have anti-MRSA activity.^64^ It was identified in a high-throughput screen for compounds that prevented MRSA from killing nematodes and permeabilized MRSA persisters.^64^ Bithionol was found to have excellent potency against gram-positive pathogens (MICs 0.5–2 μg/mL), but poor activity against gram-negative bacteria.^64^ A SYTOX Green assay shows this activity observed in both exponential and stationary phase MRSA, and includes antipersister activity.^64^ This also confirms a membrane-disrupting mechanism of action.^64^ Moreover, bithionol shows selectivity for bacterial membranes over mammalian due to the high cholesterol content of mammalian membranes.^64^ Cholesterol reduces the fluidity of human cell membranes, dissuading bithionol from penetrating.^64^ SAR studies were unable to find a more effective analogue than bithionol itself.^64^ Repurposing bithionol against MRSA proved an effective strategy to quickly identify a compound with excellent activity, particularly against persisters.^64^

In an effort to identify alternative bacterial targets, our collaborators in the Sieber lab screened 232 commercial kinase inhibitors originally intended for cancer treatment.^10^ Sorafenib shows low MICs against gram-positive bacteria, particularly methicillin-susceptible *S. aureus* (MSSA) and several MRSA strains.^10^ Unlike our bithionol investigation, SAR studies fruitfully identified a lead **PK150** with 10-fold potency over the initial hit.^10^ Chemical proteomics revealed two mechanisms: lowering menaquinone levels and dysregulating protein secretion.^10^ Both sorafenib and **PK150** were found to bind to the demethylmenaquinone methyltransferase MenG, which catalyzes the final biosynthetic step toward the essential vitamin menaquinone.^10^ This was confirmed with lowered menaquinone levels in treated cells, as well as a radioactive enzymatic assay, measuring for incorporation (or lack thereof) of a radiolabeled methyl.^10^ Sorafenib and **PK150** were found to activate signal peptidase SpsB, confirmed by significantly elevated levels of SpsB-processed proteins in the secretome.^10^

The Wu lab identified hexestrol, previously used for estrogen replacement therapy, to have gram-positive activity, including several MRSA strains, and no gram-negative activity.^65^ It was also found to eradicate and inhibit biofilm formation and showed synergy with aminoglycosides.^65^ Based on the phenolic structure, one might assume hexestrol has a membrane-based mechanism of action. However, there was no detected change in fluorescence signals with either SYTOX Green or DiSC3(5) assays, eliminating this possibility.^65^ Instead, exposure to hexestrol was found to increase ATP levels through inhibition of ATPase, altering cellular energy metabolism.^65^ This confers the compound’s synergy to aminoglycosides by increasing NADPH and proton motive force (PMF), stimulating internalization of aminoglycoside antibiotics.^65^ The activity of hexestrol, sorafenib, and its analogue strongly display the effectiveness of utilizing a drug repurposing strategy to find unique mechanisms of action. New antibacterial mechanisms are essential to defying antimicrobial resistance.

## Future Directions and Conclusions

There have been exceptional advancements in the development of MRSA compounds in the past decade. Beyond the three classes of molecules outlined herein, there have been major advancements in other anti-MRSA scaffolds such as coumarin,^66^ synthetic alkaloids,^67–69^ and disinfectants.^29,70,71^ However, the development of gram-positive antibacterial compounds is still overlooked. Although daunting, this presents a unique opportunity for further therapeutic development. For example, many compounds incorporate a dimethyl quaternary nitrogen as amphiphilic motif to increase membrane affinity. These quaternary nitrogens increase potency, but very little SAR has been conducted to expand this scope, particularly on these outlined scaffolds. Recent work in the disinfectant space has showed the alkyl length on these QACs plays a vital role in microbial activity suggesting their importance in MRSA activity.^29^ Furthermore, quaternary phosphorus compounds (QPC) developed by our lab outperformed traditional QACs.^71–74^ There are very few quaternary phosphorus motifs appended on an antibiotic scaffold, highlighting a clear area for potential development if their toxicity risk can be mitigated.

In addition to these chemical modifications, the approach used to identify novel scaffolds can also be improved. Due to the large amount of existing SAR on many of these structures, deep learning and predictive modeling are becoming common ways to identify lead analogues to synthesize. This machine learning approach can save valuable time and synthetic effort. As such, this approach gained the attention of multiple groups and is becoming a more common practice in antimicrobial drug development. The Stokes and Collins lab exemplify this strategy with the development of abaucin, an *Acinetobacter baumannii* therapeutic.^75^ In addition to traditional SAR, deep learning has also entered the drug repurposing space to aid in drug selection.^76,77^

Most of these outlined compound classes have not been investigated in combination or synergistically with current antibiotics. While honokiol and magnolol, for example, show potent synergistic effects, their synthetic analogues have not been similarly investigated. CAMPs in particular have been well studied in combination with approved antibiotics,^78^ but the above compounds featuring CAMP isosteres have not.

The use of HTS can aid in drug repurposing for antimicrobial development. Although deep learning can assist in molecule selection and repurposing *in silico*, *in vitro* testing of the actual compounds is the definitive way to determine a lead, albeit time-consuming. HTE allows for thousands of drugs to be screened and accumulate a plethora of information.^79,80^ This is not only valuable data on its own but can aid in training sets for deep learning. Another technology that has recently gained traction is direct-to-biology workflow (D2B). D2B allows for synthetic compounds to be directly tested without the need for isolation or purification. This drastically decreases the synthetic burden of a medicinal chemistry campaign and allows for a large library of analogues to be explored.^81,82^ Although D2B has seen recent success with other systems, very few examples for D2B have been reported in the antibacterial field, highlighting an area for further development.

However, all these above approaches would not be possible without adequate funding and follow-on support for commercialization. Currently, federal funding is at an all-time low and support for the NIAID, the primary funder for antibiotic research, is in peril. Continued support for both the NIH, and in particular the NIAID, is absolutely necessary. Even more concerning is the current incentive model for commercializing new antibiotics. Given our current FDA regulatory pathway, new drugs need to be extremely specific and best in class. In addition, if approved, their use will be limited and only prescribed as a last resort. Thus, there is little financial incentive for antibiotic research to be conducted in industry. As a case study, Microcide Pharmaceuticals was a company formed in the 1990s on the basis of developing novel anti-MRSA cephalosporins, but operations were discontinued by 2003.^83^ In order to address this market inefficiency, a number of congressional acts in the United States have been put forward over the past decade. The most promising is the PASTEUR (Pioneering Antimicrobial Subscriptions To End Upsurging Resistance) Act, introduced in congress in 2023. The Act would award grants and contracts to manufacturers with researched or approved antimicrobial compounds.^84^ It would also set up a subscription model of reimbursement, ensuring companies would be financially incentivized to pursue them. Nonetheless, the burden of antibiotic development typically falls on academia with most work focusing on gram-negative pathogens due to their inherent accumulation challenges. This has left a startling void in gram-positive drug discovery for the future.

Overall, the development of novel small molecules to combat gram-positive bacteria and specifically MRSA is of paramount importance. Fortunately, there have been major advancements in this field over the past decade. Despite their structural variety, each motif discussed here presents a new opportunity for combating MRSA and other gram-positive infections and contributes to the fight against increasing antibiotic resistance.

## Data Availability

The data underlying this study are available in the published article.

## References

[1] Brown E. D., Wright G. D. (2016). Antibacterial drug discovery in the resistance era. Nature.

[2] Antibiotic Resistance Threats in the United States, 2019; Centers for Disease Control, 2019; DOI: 10.15620/cdc:82532.

[3] Peacock S. J., Paterson G. K. (2015). Mechanisms of Methicillin Resistance in Staphylococcus
aureus. Annu. Rev. Biochem..

[4] Nikolic, P. ; Mudgil, P. The Cell Wall, Cell Membrane and Virulence Factors of Staphylococcus aureus and Their Role in Antibiotic Resistance. In Microorganisms, 2023; Vol. 11. 10.3390/microorganisms11020259 PMC9965861 36838224

[5] Shalaby M.-A. W., Dokla E. M. E., Serya R. A. T., Abouzid K. A. M. (2020). Penicillin binding
protein 2a: An overview and a medicinal chemistry perspective. Eur. J. Med. Chem..

[6] Eyal Z., Matzov D., Krupkin M., Paukner S., Riedl R., Rozenberg H., Zimmerman E., Bashan A., Yonath A. (2016). A novel pleuromutilin
antibacterial compound, its binding mode and selectivity mechanism. Sci. Rep..

[7] Eun Y. J., Foss M. H., Kiekebusch D., Pauw D. A., Westler W. M., Thanbichler M., Weibel D. B. (2012). DCAP: a broad-spectrum antibiotic
that targets the cytoplasmic membrane of bacteria. J. Am. Chem. Soc..

[8] Kim S. Y., Kim J., Jeong S. I., Jahng K. Y., Yu K. Y. (2015). Antimicrobial Effects
and Resistant Regulation of Magnolol and Honokiol on Methicillin-Resistant
Staphylococcus aureus. Biomed Res. Int..

[9] Tan Z., Deng J., Ye Q., Zhang Z. (2022). The Antibacterial Activity
of Natural-derived Flavonoids. Curr. Top Med.
Chem..

[10] Le P., Kunold E., Macsics R., Rox K., Jennings M. C., Ugur I., Reinecke M., Chaves-Moreno D., Hackl M. W., Fetzer C. (2020). Repurposing human kinase
inhibitors to create an antibiotic active against drug-resistant Staphylococcus
aureus, persisters and biofilms. Nat. Chem..

[11] Kavanagh F., Hervey A., Robbins W. J. (1951). Antibiotic Substances From Basidiomycetes. Proc. Natl. Acad. Sci. U. S. A..

[12] Long K. S., Poehlsgaard J., Hansen L. H., Hobbie S. N., Böttger E. C., Vester B. (2009). Single 23S rRNA mutations at the ribosomal peptidyl
transferase centre confer resistance to valnemulin and other antibiotics
in < i > Mycobacterium smegmatis</i> by perturbation of
the
drug binding pocket. Mol. Microbiol..

[13] Drews J., Georgopoulos A., Laber G., Schütze E., Unger J. (1975). Antimicrobial Activities of 81.723 hfu, a New Pleuromutilin Derivative. Antimicrob. Agents Chemother..

[14] Burch, D. G. S. ; Ripley, P. H. ; Zeisl, E. Veterinary Use of a Pleuromutilin Derivative. World Patent WO1998001127A1, 1998.

[15] Jones R. N., Fritsche T. R., Sader H. S., Ross J. E. (2006). Activity of Retapamulin
(SB-275833), a Novel Pleuromutilin, against Selected Resistant Gram-Positive
Cocci. Antimicrob. Agents Chemother..

[16] Sader H. S., Biedenbach D. J., Paukner S., Ivezic-Schoenfeld Z., Jones R. N. (2012). Antimicrobial Activity of the Investigational Pleuromutilin
Compound BC-3781 Tested against Gram-Positive Organisms Commonly Associated
with Acute Bacterial Skin and Skin Structure Infections. Antimicrob. Agents Chemother..

[17] Multi-Discipline Review and Evaluation of Lefamulin Injection and Tablets; Center for Drug Evaluation and Research, 2018; https://www.accessdata.fda.gov/drugsatfda_docs/nda/2019/211672Orig1s000,%20211673Orig1s000MultidisciplineR.pdf (accessed 2024-12-30).

[18] Murphy S.
K., Zeng M., Herzon S. B. (2017). A modular and enantioselective synthesis
of the pleuromutilin antibiotics. Science.

[19] Farney E. P., Feng S. S., Schafers F., Reisman S. E. (2018). Total Synthesis
of (+)-Pleuromutilin. J. Am. Chem. Soc..

[20] Goethe O., DiBello M., Herzon S. B. (2022). Total synthesis
of structurally diverse
pleuromutilin antibiotics. Nat. Chem..

[21] Heidtmann C. V., Voukia F., Hansen L. N., Sorensen S. H., Urlund B., Nielsen S., Pedersen M., Kelawi N., Andersen B. N., Pedersen M. (2020). Discovery of a Potent
Adenine-Benzyltriazolo-Pleuromutilin
Conjugate with Pronounced Antibacterial Activity against MRSA. J. Med. Chem..

[22] Heidtmann C. V., Fejer A. R., Staerk K., Pedersen M., Asmussen M. G., Hertz F. B., Prabhala B. K., Frimodt-Moller N., Klitgaard J. K., Andersen T. E., Nielsen C. U., Nielsen P. (2024). Hit-to-Lead
Identification and Validation of a Triaromatic Pleuromutilin Antibiotic
Candidate. J. Med. Chem..

[23] Li K., Lin C., Hu Y. H., Wang J., Jin Z., Zeng Z. L., Tang Y. Z. (2024). Design, Synthesis, Biological Evaluation, and Molecular
Docking Studies of Pleuromutilin Derivatives Containing Thiazole. ACS Infect Dis.

[24] Liu K., Xia J., Li Y., Li B. B., Wang M. Q., Zhou Q., Ma M. L., He Q. R., Yang W. Q., Liu D. F. (2024). Discovery
of Novel Coumarin Pleuromutilin Derivatives as Potent Anti-MRSA
Agents. J. Med. Chem..

[25] Xia J., Xin L., Li J., Tian L., Wu K., Zhang S., Yan W., Li H., Zhao Q., Liang C. (2023). Discovery of Quaternized
Pyridine-Thiazole-Pleuromutilin Derivatives with Broad-Spectrum Antibacterial
and Potent Anti-MRSA Activity. J. Med. Chem..

[26] Yi Y., Zhang J., Lin S., Wang H., Li G., Yang S., Shang R., Zhang R., Li F. (2025). Design, synthesis,
and biological evaluation of novel pleuromutilin derivatives with
methicillin-resistant Staphylococcus aureus -targeting phenol linker
groups. Eur. J. Med. Chem..

[27] Chai F., Wang J., Zhou K. X., Wang S. K., Liu Y. H., Jin Z., Tang Y. Z. (2022). Design,
synthesis and biological evaluation of novel
pleuromutilin derivatives possessing 4-aminothiophenol linker as promising
antibacterial agents. Bioorg Chem..

[28] Lolk L., Po̷hlsgaard J., Jepsen A. S., Hansen L. H., Nielsen H., Steffansen S. I., Sparving L., Nielsen A. B., Vester B., Nielsen P. (2008). A Click Chemistry Approach to Pleuromutilin Conjugates
with Nucleosides or Acyclic Nucleoside Derivatives and Their Binding
to the Bacterial Ribosome. J. Med. Chem..

[29] Toles Z. E.
A., Thierer L. M., Wu A., Bezold E. L., Rachii D., Sanchez C. A., Vargas-Cuebas G. G., Keller T. M., Carroll P. J., Wuest W. M. (2024). Bushy-Tailed
QACs: The Development of Multicationic
Quaternary Ammonium Compounds with a High Degree of Alkyl Chain Substitution. ChemMedChem..

[30] Zuo G. Y., Zhang X. J., Han J., Li Y. Q., Wang G. C. (2015). In vitro
synergism of magnolol and honokiol in combination with antibacterial
agents against clinical isolates of methicillin-resistant Staphylococcus
aureus (MRSA). BMC Complement Altern Med..

[31] Ochoa C., Solinski A. E., Nowlan M., Dekarske M. M., Wuest W. M., Kozlowski M. C. (2020). A Bisphenolic Honokiol Analog Outcompetes Oral Antimicrobial
Agent Cetylpyridinium Chloride via a Membrane-Associated Mechanism. ACS Infectious Diseases.

[32] Guo Y., Hou E., Wen T., Yan X., Han M., Bai L. P., Fu X., Liu J., Qin S. (2021). Development of Membrane-Active Honokiol/Magnolol
Amphiphiles as Potent Antibacterial Agents against Methicillin-Resistant
Staphylococcus aureus (MRSA). J. Med. Chem..

[33] Zhang F., Fang H., Zhao Y., Zhao B., Qin S., Wang Y., Guo Y., Liu J., Xu T. (2024). A membrane-targeting
magnolol derivative for the treatment of methicillin-resistant Staphylococcus
aureus infections. Front Microbiol.

[34] Yang R., Cui L., Xu S., Zhong Y., Xu T., Liu J., Lan Z., Qin S., Guo Y. (2024). Membrane-Targeting Amphiphilic Honokiol
Derivatives Containing an Oxazole Moiety as Potential Antibacterials
against Methicillin-Resistant Staphylococcus aureus. J. Med. Chem..

[35] Yang R., Cui L., Xu T., Zhong Y., Hu S., Liu J., Qin S., Wang X., Guo Y. (2024). Discovery of membrane-targeting amphiphilic
honokiol derivatives containing an oxazolethione moiety to combat
methicillin-resistant Staphylococcus aureus (MRSA) infections. Eur. J. Med. Chem..

[36] Solinski A. E., Ochoa C., Lee Y. E., Paniak T., Kozlowski M. C., Wuest W. M. (2018). Honokiol-Inspired Analogs as Inhibitors of Oral Bacteria. ACS Infect Dis.

[37] Lin S., Liu J., Li H., Liu Y., Chen Y., Luo J., Liu S. (2020). Development
of Highly Potent Carbazole Amphiphiles as Membrane-Targeting
Antimicrobials for Treating Gram-Positive Bacterial Infections. J. Med. Chem..

[38] Liu J., Li H., Li H., Fang S., Shi J., Chen Y., Zhong R., Liu S., Lin S. (2021). Rational Design of
Dipicolylamine-Containing Carbazole Amphiphiles Combined with Zn­(2+)
as Potent Broad-Spectrum Antibacterial Agents with a Membrane-Disruptive
Mechanism. J. Med. Chem..

[39] Verma S. K., Verma R., Kumar K. S. S., Banjare L., Shaik A. B., Bhandare R. R., Rakesh K. P., Rangappa K. S. (2021). A key review on
oxadiazole analogs as potential methicillin-resistant Staphylococcus
aureus (MRSA) activity: Structure-activity relationship studies. Eur. J. Med. Chem..

[40] Xu S., Kang A., Tian Y., Li X., Qin S., Yang R., Guo Y. (2024). Plant Flavonoids with Antimicrobial
Activity against Methicillin-Resistant Staphylococcus aureus (MRSA). ACS Infect Dis.

[41] Liang M., Ge X., Xua H., Ma K., Zhang W., Zan Y., Efferth T., Xue Z., Hua X. (2022). Phytochemicals with
activity against methicillin-resistant Staphylococcus aureus. Phytomedicine.

[42] Biharee A., Sharma A., Kumar A., Jaitak V. (2020). Antimicrobial flavonoids
as a potential substitute for overcoming antimicrobial resistance. Fitoterapia.

[43] Cushnie T. P., Lamb A. J. (2011). Recent advances
in understanding the antibacterial
properties of flavonoids. Int. J. Antimicrob.
Agents.

[44] Cheng W., Xu T., Cui L., Xue Z., Liu J., Yang R., Qin S., Guo Y. (2023). Discovery of Amphiphilic Xanthohumol Derivatives as
Membrane-Targeting Antimicrobials against Methicillin-Resistant Staphylococcus
aureus. J. Med. Chem..

[45] Yang R., Cheng W., Huang M., Xu T., Zhang M., Liu J., Qin S., Guo Y. (2024). Novel membrane-targeting
isoxanthohumol-amine
conjugates for combating methicillin-resistant Staphylococcus aureus
(MRSA) infections. Eur. J. Med. Chem..

[46] Moldovan C. V., Mantea L. E., Savu M., Jones P. G., Sarbu L. G., Stefan M., Birsa M. L. (2024). Novel Tricyclic
Flavonoids as Promising
Anti-MRSA Agents. Pharmaceuticals (Basel).

[47] Wei M. Z., Zhu Y. Y., Zu W. B., Wang H., Bai L. Y., Zhou Z. S., Zhao Y. L., Wang Z. J., Luo X. D. (2024). Structure
optimizing of flavonoids against both MRSA and VRE. Eur. J. Med. Chem..

[48] Flemetakis A. C., Tsambaos D. G. (1989). Effects of Synthetic
Retinoids on the Growth of Bacteria
and Their Susceptibility to Antibiotics. Journal
of Chemotherapy.

[49] Rusu A., Tanase C., Pascu G.-A., Todoran N. (2020). Recent Advances Regarding
the Therapeutic Potential of Adapalene. Pharmaceuticals.

[50] Pechère M., Germanier L., Siegenthaler G., Pechère J. C., Saurat J. H. (2002). The Antibacterial
Activity of Topical Retinoids: The
Case of Retinaldehyde. Dermatology.

[51] Kim W., Zhu W., Hendricks G. L., Van Tyne D., Steele A. D., Keohane C. E., Fricke N., Conery A. L., Shen S., Pan W. (2018). A new
class of synthetic retinoid antibiotics effective against bacterial
persisters. Nature.

[52] André C., Schrank C. L., Cheng Jaramillo A. V., Mylonakis E., Wuest W. M., Gilmore M. S., Kim W., Bispo P. J. M. (2023). Antimicrobial
activity of a new class of synthetic retinoid antibiotics and comparator
agents against ocular staphylococci. Frontiers
in Antibiotics.

[53] Xie Y. P., Sangaraiah N., Meng J. P., Zhou C. H. (2022). Unique Carbazole-Oxadiazole
Derivatives as New Potential Antibiotics for Combating Gram-Positive
and -Negative Bacteria. J. Med. Chem..

[54] Cheng C. Y., Chang C. P., Lauderdale T. Y., Yu G. Y., Lee J. C., Jhang Y. W., Wu C. H., Ke Y. Y., Sadani A. A., Yeh C. F. (2016). Bromomethylthioindole
Inspired Carbazole Hybrids as
Promising Class of Anti-MRSA Agents. ACS Med.
Chem. Lett..

[55] Viering B., Cunningham T., King A., Blackledge M. S., Miller H. B. (2022). Brominated Carbazole with Antibiotic Adjuvant Activity
Displays Pleiotropic Effects in MRSA’s Transcriptome. ACS Chem. Biol..

[56] Berndsen R., Cunningham T., Kaelin L., Callender M., Boldog W. D., Viering B., King A., Labban N., Pollock J. A., Miller H. B. (2022). Identification and Evaluation
of Brominated Carbazoles as a Novel Antibiotic Adjuvant Scaffold in
MRSA. ACS Med. Chem. Lett..

[57] Xie Y. P., Ansari M. F., Zhang S. L., Zhou C. H. (2021). Novel carbazole-oxadiazoles
as potential Staphylococcus aureus germicides. Pestic. Biochem. Physiol..

[58] Maneerat W., Ritthiwigrom T., Cheenpracha S., Promgool T., Yossathera K., Deachathai S., Phakhodee W., Laphookhieo S. (2012). Bioactive
carbazole alkaloids from Clausena wallichii roots. J. Nat. Prod.

[59] Zhang F.-F., Gan L.-L., Zhou C.-H. (2010). Synthesis,
antibacterial and antifungal
activities of some carbazole derivatives. Bioorg.
Med. Chem. Lett..

[60] Adeniyi E. T., Kruppa M., De Benedetti S., Ludwig K. C., Krisilia V., Wassenberg T. R., Both M., Schneider T., Müller T. J. J., Kalscheuer R. (2024). Synthesis of Bisindole Alkaloids
and Their Mode of Action against Methicillin-Resistant Staphylococcus
Aureus. ACS Infectious Diseases.

[61] Reinhardt T., Lee K. M., Niederegger L., Hess C. R., Sieber S. A. (2022). Indolin-2-one
Nitroimidazole Antibiotics Exhibit an Unexpected Dual Mode of Action. ACS Chem. Biol..

[62] Zeczycki T. N., Milton M. E., Jung D., Thompson R. J., Jaimes F. E., Hondros A. D., Palethorpe S., Melander C., Cavanagh J. (2022). 2-Aminoimidazole
Analogs Target PhoP Altering DNA Binding Activity and Affect Outer
Membrane Stability in Gram-Negative Bacteria. Biochemistry.

[63] Kim W., Steele A. D., Zhu W., Csatary E. E., Fricke N., Dekarske M. M., Jayamani E., Pan W., Kwon B., Sinitsa I. F. (2018). Discovery and Optimization of nTZDpa as an
Antibiotic Effective Against Bacterial Persisters. ACS Infectious Diseases.

[64] Kim W., Zou G., Hari T. P. A., Wilt I. K., Zhu W., Galle N., Faizi H. A., Hendricks G. L., Tori K., Pan W., Huang X., Steele A. D., Csatary E. E., Dekarske M. M., Rosen J. L., Ribeiro N. d. Q., Lee K., Port J., Fuchs B. B., Vlahovska P. M., Wuest W. M., Gao H., Ausubel F. M., Mylonakis E. (2019). A selective membrane-targeting repurposed
antibiotic with activity against persistent methicillin-resistant
< i > Staphylococcus aureus</i>. Proc.
Natl. Acad. Sci. U. S. A..

[65] Liu S., She P., Li Z., Li Y., Li L., Yang Y., Zhou L., Wu Y. (2023). Antibacterial
and antibiofilm efficacy
of repurposing drug hexestrol against methicillin-resistant Staphylococcus
aureus. Int. J. Med. Microbiol.

[66] Yang R., Xue Z., Li X., Xu T., Zhong Y., Hu S., Qin S., Guo Y. (2024). Novel natural osthole-inspired amphiphiles as membrane
targeting antibacterials against methicillin-resistant Staphylococcus
aureus (MRSA). Eur. J. Med. Chem..

[67] Liu Z. H., Wang W. M., Zhang Z., Sun L., Wu S. C. (2022). Natural
Antibacterial and Antivirulence Alkaloids From Macleaya cordata Against
Methicillin-Resistant Staphylococcus aureus. Front Pharmacol.

[68] Herrera K. M. S., Lopes G. F. M., Oliveira M. E., Sousa J. F., Lima W. G., Silva F. K., Brito J. C. M., Gomes A. J. P. S., Viana G. H. R., Soares A. C. (2022). A 3-alkylpyridine-bearing
alkaloid exhibits potent antimicrobial activity against methicillin-resistant
Staphylococcus aureus (MRSA) with no detectable resistance. Microbiological Research.

[69] Wu K.-Y., Yao F.-H., Ren X.-M., Hang X.-D., Bai Y.-F., Qi S.-H. (2025). Multi-target anti-MRSA mechanism
and antibiotic synergistic effect
of marine alkaloid Ascomylactam A in vitro and in vivo against clinical
MRSA strains. Biochem. Pharmacol..

[70] Sanchez C. A., Vargas-Cuebas G. G., Michaud M. E., Allen R. A., Morrison-Lewis K. R., Siddiqui S., Minbiole K. P. C., Wuest W. M. (2024). Highly Effective
Biocides against Pseudomonas aeruginosa Reveal New Mechanistic Insights
Across Gram-Negative Bacteria. ACS Infectious
Diseases.

[71] McDonough D., Sanchez C. A., Wuest W. M., Minbiole K. P. C. (2025). Recent
developments
in antimicrobial small molecule quaternary phosphonium compounds (QPCs)–synthesis
and biological insights. RSC Medicinal Chemistry.

[72] Brayton S.
R., Toles Z. E. A., Sanchez C. A., Michaud M. E., Thierer L. M., Keller T. M., Risener C. J., Quave C. L., Wuest W. M., Minbiole K. P. C. (2023). Soft
QPCs: Biscationic Quaternary Phosphonium Compounds
as Soft Antimicrobial Agents. ACS Infectious
Diseases.

[73] Thierer L. M., Petersen A. A., Michaud M. E., Sanchez C. A., Brayton S. R., Wuest W. M., Minbiole K. P. C. (2023). Atom Economical
QPCs: Phenyl-Free
Biscationic Quaternary Phosphonium Compounds as Potent Disinfectants. ACS Infectious Diseases.

[74] Sommers K. J., Michaud M. E., Hogue C. E., Scharnow A. M., Amoo L. E., Petersen A. A., Carden R. G., Minbiole K. P. C., Wuest W. M. (2022). Quaternary
Phosphonium Compounds: An Examination of Non-Nitrogenous Cationic
Amphiphiles That Evade Disinfectant Resistance. ACS Infectious Diseases.

[75] Liu G., Catacutan D. B., Rathod K., Swanson K., Jin W., Mohammed J. C., Chiappino-Pepe A., Syed S. A., Fragis M., Rachwalski K. (2023). Deep learning-guided discovery of an antibiotic
targeting Acinetobacter baumannii. Nat. Chem.
Biol..

[76] Pham T.-H., Qiu Y., Zeng J., Xie L., Zhang P. (2021). A deep learning framework
for high-throughput mechanism-driven phenotype compound screening
and its application to COVID-19 drug repurposing. Nature Machine Intelligence.

[77] Huang K., Chandak P., Wang Q., Havaldar S., Vaid A., Leskovec J., Nadkarni G. N., Glicksberg B. S., Gehlenborg N., Zitnik M. (2024). A foundation model
for clinician-centered
drug repurposing. Nature Medicine.

[78] Taheri-Araghi S. (2024). Synergistic
action of antimicrobial peptides and antibiotics: current understanding
and future directions. Frontiers in Microbiology.

[79] Di
Bonaventura G., Lupetti V., Di Giulio A., Muzzi M., Piccirilli A., Cariani L., Pompilio A. (2023). Repurposing
High-Throughput Screening Identifies Unconventional Drugs with Antibacterial
and Antibiofilm Activities against Pseudomonas aeruginosa under Experimental
Conditions Relevant to Cystic Fibrosis. Microbiology
Spectrum.

[80] Khambhati, K. ; Siruka, D. ; Ramakrishna, S. ; Singh, V. Current progress in high-throughput screening for drug repurposing. In Progress in Molecular Biology and Translational Science; Singh, V. , Ed.; Academic Press, 2024; Vol. 205, Chapter 10, pp 247–257. 10.1016/bs.pmbts.2024.03.013 38789182

[81] Wang Z., Shaabani S., Gao X., Ng Y. L. D., Sapozhnikova V., Mertins P., Krönke J., Dömling A. (2023). Direct-to-biology,
automated, nano-scale synthesis, and phenotypic screening-enabled
E3 ligase modulator discovery. Nat. Commun..

[82] Hendrick C. E., Jorgensen J. R., Chaudhry C., Strambeanu I. I., Brazeau J.-F., Schiffer J., Shi Z., Venable J. D., Wolkenberg S. E. (2022). Direct-to-Biology Accelerates PROTAC
Synthesis and
the Evaluation of Linker Effects on Permeability and Degradation. ACS Med. Chem. Lett..

[83] Barrett J. F. (2005). Can biotech
deliver new antibiotics?. Curr. Opin. Microbiol..

[84] S.1355, PASTEUR Act of 2023, 118th Congress (2023–2024); U.S. Congress, 2023; https://www.congress.gov/bill/118th-congress/senate-bill/1355#:~:text=Introduced%20in%20Senate%20(04%2F27%2F2023)&text=This%20bill%20authorizes%20the%20Department,and%20contains%20other%20related%20provisions.

